# Chitinase improves the available energy, amino acids digestibility of black soldier fly and fecal microbiota of growing pigs

**DOI:** 10.5713/ab.24.0920

**Published:** 2025-04-11

**Authors:** Yuansen Yang, Shenrui Xu, Wenjun Gao, Yu Wei, Lu Wang, Changhua Lai

**Affiliations:** 1 State Key Laboratory of Animal Nutrition and Feeding, Ministry of Agriculture and Rural Affairs Feed Industry Centre, China Agriculture University, Beijing, China; 2 National Laboratory of Biomacromolecules, CAS Center for Excellence in Biomacromolecules, Institute of Biophysics, Chinese Academy of Sciences, Beijing, China

**Keywords:** Amino Acids Digestibility, Black Soldier Fly, Chitinase, Energy Value, Fecal Microbiota, Growing Pigs

## Abstract

**Objective:**

The main objective of this study was to determine the energy value and digestibility of amino acids in growing pigs fed black soldier fly (BSF) with chitinase supplementation and its effect on fecal microbiota.

**Methods:**

In Exp. 1, eighteen barrows with initial body weight (BW) of 31.6±1.2 kg were randomly assigned to 3 treatments to determine the digestible energy (DE), metabolizable energy (ME), and nutrient digestibility, including the corn-soybean meal basal group, the BSF (14.6%) with 1,500 mg/kg or without chitinase groups. Fresh feces were collected on day 10 of feeding for analysis of microbial diversity. In Exp. 2, six barrows (36.7±4.2 kg BW) with ileal T-cannulas were arranged in a 6×3 Youden square design, pigs were allotted to 3 dietary treatments to determine the digestibility of the amino acids, including the nitrogen-free group, the BSF with or without chitinase groups.

**Results:**

Supplemental chitinase improved (p<0.05) the apparent total tract digestibility (ATTD) of crude protein and dry matter in the BSF diet, improved (p<0.05) the ATTD of crude protein, ether extract and acid detergent fiber in the BSF, and increased the BSF's DE and ME by 2.34 MJ/kg and 1.37 MJ/kg, respectively. Chitinase supplementation improved (p<0.05) the composition of fecal microbiota of growing pigs, promoted (p<0.05) the colonisation of *Bacilli*, *Lactobacillaceae*, and *Lactobacillales*, and inhibited (p<0.05) the proliferation of pathogenic bacteria *Sarcina*. Adding chitinase enhanced the apparent ileal digestibility and standardized ileal digestibility of most amino acids in BSF, such as methionine, leucine, and glutamate (p<0.05).

**Conclusion:**

Adding chitinase can increase the effective energy value and amino acid ileal digestibility of BSF, regulate the composition of fecal microbiota and improve the intestinal health of growing pigs.

## INTRODUCTION

Black soldier fly (BSF), mealworm larvae, and crickets have been investigated as protein ingredients to be used as an alternative source of nutrients in poultry and swine feeds because they contain nearly 100% edible protein [1]. Compared to mealworms and crickets, BSF has a higher feed conversion rate and survival rate, and more stable nitrogen and phosphorus composition [2]. The larvae of BSF are a great source of protein and fat with 30% to 52% crude protein (CP) and 29.4% to 50.1% ether extract (EE), and the defatted BSF meal even with 56.9% CP and 4.6% EE [3–5]. Additionally, except to methionine and lysine, BSF has an essential amino acid (AA) pattern similar to fish meal, the concentration of the AAs such as methionine, lysine, tryptophan, threonine, cysteine, and valine is comparable to soybean meal, and BSF are a rich source of bioavailable arginine [4].

Chitin, also known as chitosan, is mainly found in the epidermis of BSF, pupal shells, and adult exoskeleton. The content of chitin varies in different developmental stages of the BSF. Chitin is an indigestible nitrogenous fiber that can only be broken down by the chitinase. A high chitin and chitin derivative chitosan content in the diet negatively affects nutrient utilization [6,7]. Kim et al [8] found that *in vitro* ileal digestibility of CP and *in vitro* total tract digestibility of dry matter (DM) in defatted BSF were less than those in fish meal because of chitin. Marono et al [7] found a negative correlation between *in vitro* nutrient digestibility and chitin in insects, it is unclear whether we can improve nutrient digestibility in insects by degrading chitin, we can improve the *in vivo* nutrient digestibility of insect by adding chitinase. The enzymatic product of chitin is chitooligosaccharides, it has antimicrobial properties and bacteriostatic effects on diverse, harmful, gram-negative bacteria and positive effects on the growth of beneficial microbes while increasing nutrient digestibility of the diets in weaned pigs [9,10]. Therefore, we suppose that adding exogenous chitinase can obtain the enzymatic hydrolysis product chitooligosaccharides, improving intestinal health by regulating pigs’ intestinal microbiota. Thus, we hypothesized that adding chitinase to feed can improve the nutritional value of BSF, such as by increasing the energy value and nutrient digestibility, while exploring the effects of chitinase on fecal microbiota. The aim of the present study was to evaluate the effect of chitinase on energy value and the nutrients digestibility of BSF, and feacl microbiota for growing pigs (Exp. 1) and the apparent/standardized ileal digestibility of protein and AA in growing pigs (Exp. 2).

## MATERIALS AND METHODS

### Animal care

The protocols of these two experiments were conducted at the Dabeinong (Yutian) Swine Science Laboratory Center and approved by the Institutional Animal Care and Use Committee of China Agricultural University (No.AW80904202-1-2). Full fat BSF powder was obtained from Beijing Dabeinong Science and Technology Group Co., Ltd. (Beijing, China). The enzyme activity of chitinase was 2000,000 U/g, purchased from Fuyuan Biotechnology Co., Ltd. (Fuzhou, China) Chitinase was added at a rate of 1,500 mg/kg, and the same batch of BSF and chitinase was used in all experiments.

### Animals, dietary treatments and experimental design

In Exp 1, Eighteen Duroc×Landrace×Yorkshire crossbred barrows (body weight [BW] 31.57±1.24 kg) were randomly divided into 3 treatment groups with six replicates, including the corn-soybean basal diet group, and the BSF group without or with 1,500 mg/kg chitinase addition groups. Two BSF groups contained 15% BSF, which replaced 15% of the energy-supplying ingredients in the diet (inclued corn and soybean meal). The nutrient compositions of the experimental diets (Table 1) conformed to the nutritional requirements of pigs as recommended by NRC [11]. The analyzed composition of the experimental diets and BSF are presented in Table 2.

In Exp 2, Six Duroc×Landrace×Yorkshire crossbred barrows (BW 36.72±4.2 kg) fitted with T-cannulas at the distal ileum as the method of Stein et al [12]. In a 6×3 Youden square design, pigs were allotted to 3 dietary treatments in 3 collection periods. Each experiment period lasted 7 days, with the first 5 days being the acclimation period and the last 2 days being the digestive collection period. The experimental diets included an nitrogen (N)-free diet was made to calculate the endogenous AA losses, and 20% of BSF as the sole AA source was contained in the BSF with or without chitinase diets. Chromic oxide (3 g/kg) was used in all diets as an indigestible marker. The analyzed AA composition of BSF, and experimental diets is shown in Table 3.

### Feeding and sample collection

In Exp 1, All pigs were housed individually in stainlesssteel metabolism cages (1.4 m×0.7 m×0.6 m) at a temperature of 22±2°C and had free access to water. The feed supply was equal to 4% of BW and was divided equally into 2 parts and fed at 08:30 and 15:30 every day. The feed intake was recorded every feeding time, and the whole experiment lasted 12 days with the first 7 d as an acclimation period and the last 5 d of feces and urine collection. The feces were collected in plastic bags when they appeared and stored at −20°C. Buckets with 10 mL of 6 mol/L HCl per 1,000 mL were used to collect urine. The urine volume was recorded daily, seal 10% of the urine in a polyethylene screw cap bottle and stored at −20°C. Finally, the feces and urine of each experimental pig were separately mixed and then 300 g feces and 45 mL urine were taken as subsamples. Before analysis, fecal subsamples were dried for 72 h in a 65°C drying oven (B0D-150-ll; BIOBASE, Shangdong, China) and ground through a 1 mm screen (FW-100; Beijing Ever Bright Medical Treatment Instrment Co., Ltd, Beijing, China). A 5 mL fresh fecal samples of the eighteen pigs were collected by rectal stimulation on day 10 of feeding, the fecal samples were stored in cryosurgery tubes, immediately deep-frozen in liquid nitrogen, and stored at −80°C to be used for subsequent sequencing of microbial diversity by 16S rRNA.

In Exp 2, After two weeks recovery period, the pig was weighed and housed individually in stainlesssteel metabolism cages described in Exp 1. Plastic bags were used to collect the digesta from 08:00 to 17:00, the digesta were removed to −20°C refrigerator whenever the digesta were full or at least every 30 min. At the end of the experiment, digesta sample per pig was thawed, mixed, subsampled, and then lyophilized in a vacuum freeze-dryer (Tofon Freeze Drying Systems, Shanghai, China) and ground through a 1 mm screen for further chemical analysis.

### Chemical analysis

The DM content according to AOAC method 930.15, CP content following AOAC method 984.13, EE content according to AOAC method 920.39), ash content according to AOAC method 942.05, calcium according to AOAC method 968.08, phosphorus according to AOAC method 964.06 [13]. CP in BSF sources was calculated by multiplying N by 5.60 [14]. The gross energy (GE) of diets, feces, and urine was measured by an automatic isoperibol oxygen bomb calorimeter (Parr 6300 calorimeter; Parr Instrument Company, Moline, IL, USA). To prepare urine samples for GE analysis, an absorption method was used to convert liquid urine into a solid form suitable for bomb calorimetry. A pre-weighed filter paper was used as the absorption medium. A fixed volume of urine was evenly applied to the absorption material, ensuring complete absorption. The samples were then placed in a 60°C to 70°C drying oven until constant weight was achieved. The dried urine samples were analyzed using a bomb calorimeter to determine their GE content. The detailed analysis process of AA and chromium content was described by Pan et al [15] and Williams et al [16], respectively. The neutral detergent fiber (NDF) and acid detergent fiber (ADF) content were determined by the procedures of Van Soest et al [17].

The microbiota community in feces was also analyzed according to Ma et al [18]. Briefly, after genomic DNA extraction using the QIAGEN QIAamp PowerFecal Pro DNA kit (QIAGEN, Germany), 1% agarose gel (Thermo Scientific, Waltham, MA, USA) was used to detect the extracted genomic DNA. The primers, V338F (50-ACTCCTACGGGAGGCAGCAG-30) and V806R (50-GGACTACHVGGGTWTCTAAT-30) targeting variable region V3 to V4 were put into use [19]. The library was constructed using NEB Next Ultra DNA Library Prep kit, and the constructed library was detected and quantified by Q-PCR using Agilent 5400; after the library was qualified, NovaSeq 6000 was used for online sequencing. The QIIME tools import plug-in was used to import the original sequence fastq file into a file format that could be processed by QIIME2. The QIIME2 dada2 plug-in was then used for quality control, trimming, denoising, splicing, and chimera removal to obtain the final feature sequence table. Next, the QIIME2 feature-classifier plug-in was applied to compare the representative sequences of amplicon sequence variants to the pre-trained GREENGENES database of version 13_8 with 99% similarity (the database was pruned to the region of V3V4 based on the 341F/806R primer pairs), and the taxonomic information table of the species was obtained. Analysis of Composition of Microbiomes, analysis of variance (ANOVA) Kruskal Wallis, linear discriminant analysis effect size (LEfSe), and DEseq2 were employed to identify bacteria that differed in abundance between groupings and samples, followed by calculation of the diversity matrix using the QIIME2 core-diversity plug-in. Characteristic sequence level Alpha diversity indices, including observed operational taxonomic unit (OTU)s, Chao1, Shannon’s index, and Faith’s phylogenetic diversity index were used to assess the degree of diversity of the samples themselves. Beta diversity indices, including Bray Curtis, unweighted UniFrac, and weighted UniFrac indices were used to assess the variability in microbial community structure between samples and were subsequently visualized using principal coordinates analysis (PCoA) and neuromuscular disease plots.

### Calculation

In Exp.1, the method of Adeole [20] was used to determine the digestible energy (DE) and metabolizable energy (ME) of 3 diets as follows:


$$\begin{array}{l} {\text{DE}}_{\text{d}}=({\text{GE}}_{\text{i}}-{\text{GE}}_{\text{f}})\times {\text{F}}_{\text{i}}\\ {\text{DE}}_{\text{f}}=[{\text{DE}}_{\text{d}}-(100\%-\text{X}\%)\times {\text{DE}}_{\text{d}}]/\text{X}\%/0.97\\ {\text{ME}}_{\text{d}}=({\text{GE}}_{\text{i}}-{\text{GE}}_{\text{F}}-{\text{GE}}_{\text{u}})\times {\text{F}}_{\text{i}}\\ {\text{ME}}_{\text{f}}=[{\text{ME}}_{\text{d}}-(100\%-\text{X}\%)\times {\text{DE}}_{\text{d}}]/\text{X}\%/0.97 \end{array}$$

in which the GE_i_, GE_f_, GE_u_ and F_i_ represent the total GE intake, the total GE content in feces and urine, and the total feed intake over the 5 d collection period, respectively. The DE_d_ and DE_f_ represent the DE values in diet and BSF with or without chitinase diets, ME_d_ and ME_f_ represent the ME values in diet and BSF with or without chitinase diets, respectively. The X% is the percentage of energy supplied by BSF with or without chitinase in the basal diet.

In Exp.2, the method of Stein et al [21] was used to determine the standard ileal digestibility (SID) of AA:


$$\begin{array}{l} \text{Apparent ileal digestibility }(\text{AID})\;(\%)=\\ {}[1-({\text{AA}}_{\text{digesta}}/{\text{AA}}_{\text{diet}})\times ({\text{Cr}}_{\text{diet}}/{\text{Cr}}_{\text{digesta}})]\times 100\\ {\text{IAA}}_{\text{end}}=({\text{AA}}_{\text{digesta}})\times ({\text{Cr}}_{\text{diet}}/{\text{Cr}}_{\text{digesta}})\\ \text{SID }(\%)=\text{AID}+({\text{IAA}}_{\text{end}}/{\text{AA}}_{\text{diet}})\times 100 \end{array}$$

where AID is apparent ileal digestibility; SID is standardized ileal digestibility. AA_diet_ and AA_digesta_ are AA concentrations of the diet and ileal digesta, respectively (g/kg of DM); Cr_diet_ and Cr_digesta_ are chromium concentrations of the diet and ileal output, respectively (g/kg of DM); and IAA_end_ refers to the basal ileal endogenous loss of an AA (g/kg of DM intake).

### Statistical analysis

In Exp. 1, the apparent total tract digestibility (ATTD) of nutrients and the DE and ME values were analyzed using one-way ANOVA of SPSS (version 26.0), multiple comparisons were performed using the LSD method. In Exp. 1 and 2, the ATTD of nutrients and AID and SID of AAs for BSF with or without chitinase were analyzed using Student’s t-test of SPSS (version 26.0), and the results of the analyzed data were expressed using the mean and the standard error of the mean, with p<0.05 indicating a significant difference in the results. The diversity of microbial flora was analyzed using R software, and Kruskal-Wallis analysis was performed to analyze the abundance of different bacteria in each group at phylum, family, and genus levels. Microbial taxonomic annotated OTU data with linear discriminant analysis greater than 2 were regarded as groups with differences in abundance and represented by their effect size plots (LEfSe). The microorganisms that differed in the abundance of fecal flora between the two treatment groups were analyzed using Welch’s t-test. Significant differences among groups were defined as p<0.05.

## RESULTS

### Chemical composition of BSF

The GE, DM, Ash, CP, EE, NDF, ADF, Ca, and total phosphorus content of the BSF were 25.38 MJ/kg, 96.40%, 10.20%, 30.91%, 40.26%, 35.41%, 19.78%, 2.80%, and 0.60%, respectively.

### Chitinase improving energy concentration and apparent total tract digestibility of nutrients in Exp.1

Table 4 shows that the supplementation of chitinase significantly improved (p<0.05) the ATTD of CP and DM in BSF diet. Table 5 shows that chitinase supplementation increased the DE and ME of BSF (p<0.01), after adding chitinase, the DE and ME of the BSF for pigs were 17.37 MJ/kg and 16.34 MJ/kg., and significantly improved the ATTD of CP (p<0.01), EE (p<0.05), and ADF (p<0.05) in BSF.

### Chitinase improving crude protein and amino acid digestibility of the black soldier fly

The effects of chitinase on AID of AA are showed in Table 6 (Exp.2). The AID of AA of BSF varies from 36.49% to 75.27%. The supplementation of chitinase significantly increased (p<0.05) the AID of methionine, leucine, phenylalanine, isoleucine, arginine, alanine, glutamic, and proline in BSF, after adding chitinase, the AID were 75.46%, 75.63%, 77.01%, 71.64%, 76.36%, 67.66%, 72.35%, and 46.45%, respectively, the average AID of essential AAs was increased by 3.53% with adding chitinase. The SID of AA of BSF varies from 45.49% to 87.64%. Chitinase supplementation significantly improved (p<0.05) the SID of methionine, tryptophan, leucine, histidine, and glutamic, after adding chitinase, the SID 85.85%, 84.00%, 85.71%, 80.64%, and 82.56%, respectively. (Table 7), the addition of chitinase increased the average SID of essential AAs in BSF by 5.19%.

### Effect of dietary addition of chitinase to black soldier fly on fecal microbiota in growing pigs

**Figure 1:**
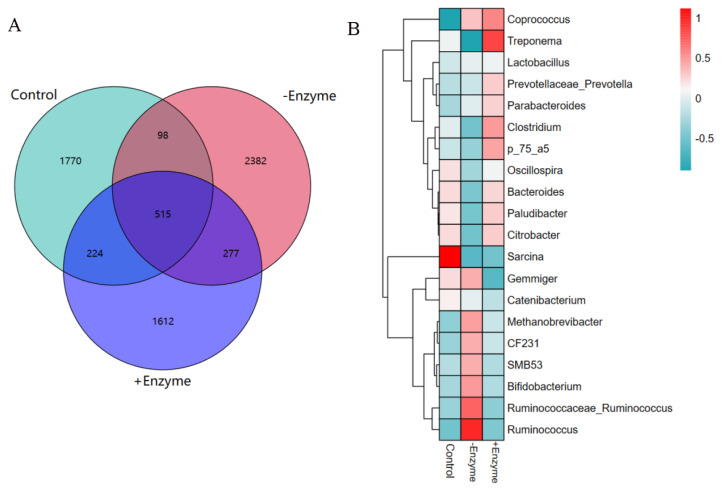
Effect of the chitinase on the abundance of flora in faeces of growing pigs (n = 6) in the diets of BSF. (A) OTU Venn diagram. (B) Genus level microbial composition heat map. OTU, operational taxonomic unit.

**Figure 2:**
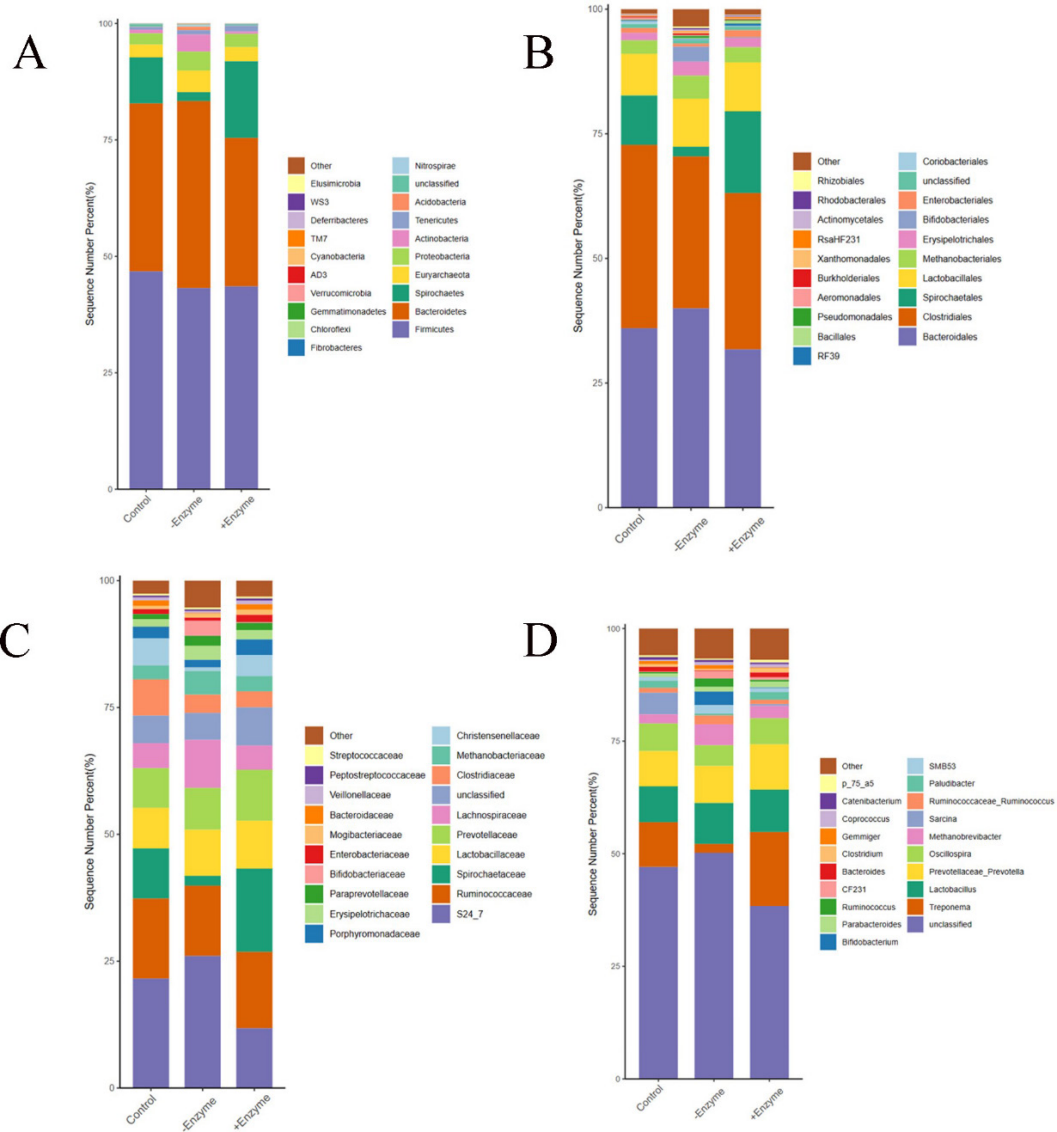
Effect of chitinase on microbial composition in faeces of growing pigs fed with BSF. (A) Microbial composition analysis at the phylum level. (B) Microbial composition analysis at the order level. (C) Microbial composition analysis at the family level. (D) Microbial composition analysis at the genus level. BSF, black soldier fly.

**Figure 3:**
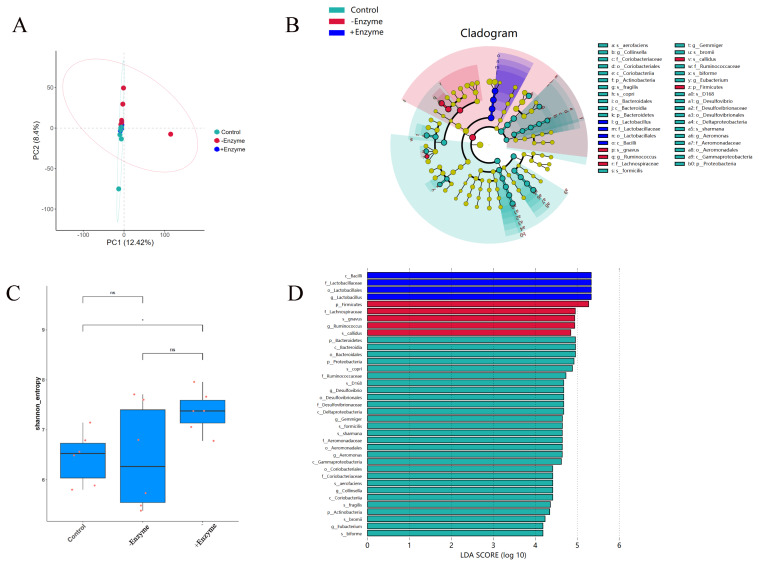
Different treatments of (A) PCoA principal component analysis of black gadfly diets with and without chitinase addition. (B,D) LEfSe multilevel species difference analysis. (C) Shannon index. PCoA, principal coordinates analysis; LEfSe, linear discriminant analysis effect size.

The Venn diagram showed 2607, 3272, and 2628 OTU were isolated from the basal, the BSF with or without chitinase groups, with 515 OTU being common (Figure 1A). At the genus level, the abundance of the top 20 bacterial communities was presented in the heatmap (Figure 1B), adding chitinase was significantly (p<0.05) decreased the *Sarcina* abundance in fecal samples from the pigs. At the phylum level, chitinase supplementation significantly (p<0.05) improved the abundance of *Acidobacteria* (Figure 2B). Down to the family level (Figure 2C), the supplementation of chitinase significantly increased (p<0.05) the abundance of *Spirochaetaceae*. At the order level (Figure 2D), the *Erysipelotrichales* abundance was higher (p<0.05) in BSF with chitinase group than that in the basal diet group. According to the PCoA based on Bray Curtis, no discernible differences at the OTU level in fecal microbiota of pigs from all treatments were found. (Figure 3A). The results of alpha diversity analysis showed that the chitinase supplementation significantly improved (p<0.05) Shannon index (Figure 3C). The specific bacterial taxa associated with treatments were identified by LEfSe analysis, the results showed that chitinase supplementation significantly (p<0.05) improved the abundance of the *Bacilli*, *Lactobacillaceae*, and *Lactobacillales* (Figures 3B, 3D).

## DISCUSSION

Spranghers et al [22] and Surendra et al [4] reported that the CP content of BSF varies from 30 to 52%, the CP content of BSF was 30.91% in this study, the variation in CP content may be due to the type of organic substrate feed to the larvae [22]. The content of nutrients in the BSF tends to show differences because of different feeding materials, and there is a positive relationship between them, and the use of substrates with higher CP content to feed BSF also has higher CP content [22]. In this experiment, the larvae was fed with restaurant waste, which generally has a slightly lower CP content than fruit waste substrates and Brewery by-product. Therefore, this may explain the lower CP content of BSF in this study Shumo et al [23] and Rawski et al [3] reported that the EE content of BSF varies from 29.4% to 51.5%, similar to the EE content of 40.26% found in the present experiment. The nutrient of BSF varies depending on the quality and quantity of food they consume. A study have shown that BSF-fed restaurant waste tend to have higher EE content, because restaurant waste has a higher oil and grease content than regular organic waste [22]. BSF is rich in essential AAs, and the AA profile in BSF is not greatly influenced by the AA profile of the substrate [22]. In this study, the lysine and methionine contents of BSF were 2.14% and 0.59%, respectively, which is consistent with previous reports [1,5]. The ash, NDF and ADF content of the BSF were 10.20%, 35.41% and 19.78% in current study, which is consistent with previous studies [4,8,24,25], however, the variation of these content is also relatively high, it may also be due to the type of organic substrate, growth stage and the processing method. At the same time, BSF is rich in both Ca, P and GE, it has the potential to be a high quality pig feed.

*In vitro*, broiler, and atlantic cod studies, it was found that a high chitin and chitin derivative chitosan content in the diet negatively affects energy value and nutrient utilization [5,8,26]. Therefore, in this study, we added chitinase to the BSF diet to target degradation of the specific chitin in the BSF. The results showed that the DE and ME of BSF were 15.03 MJ/kg and 14.97 MJ/kg, respectively, similar to the energy of soybean meal (DE 15.40 MJ/kg), and fish meal (ME 14.76 MJ/kg) [11]. There are few studies on the determination of DE and ME of BSF. Crosbie et al [27] reported that the DE and ME of full-fat BSF were 20.62 MJ/kg and 19.11 MJ/kg, respectively, which were slightly higher than our results, the reason may be related to the EE content. With supplementing chitinase, BSF's DE and ME values have significantly improved, increasing by 2.34 MJ/kg and 1.37 MJ/kg, respectively. Chitin is analyzed as the ADF fraction due to the structural similarity between chitin and cellulose [28], whereas fiber are often considered to affect energy and nutrient digestibility negatively [29]. Considering that chitin is structurally similar to ADF and that ADF fractions have been shown to contain nitrogen, as well as the fact that previous studies have found similar levels of ADF and chitin [8], we considered that we used ADF values as an indirect indicator of fiber/chitin content. The significant increase in ADF digestibility of the BSF diet after chitinase supplementation demonstrates the enzyme’s ability to degrade chitin in BSF. The significant increase in ADF digestibility of the BSF diet after chitinase supplementation demonstrates the enzyme's ability to degrade chitin in BSF. Therefore, when supplemented with chitinase, chitin is broken down, and the substances encapsulated are released, which improves the digestibility of the nutrients, such as EE, and results in a higher energy value of the BSF. This is consistent with our finding that adding chitinase significantly increased EE and ADF digestibility in BSF. Soybean meal and BSF meal have similar ATTD of CP, as reported by Newton [30]. Marono et al [7] indicated that chitin is the main factor affecting the *in vitro* protein digestibility of BSF meal for a single-stomached animal and showed that CP digestibility was negatively correlated to the chitin content. Moreover, it has been shown that high concentrations (up to 45%) of the chitin present in the cuticular exoskeleton of insects negatively affect feed intake and reduce CP digestibility [31]. With supplementing chitinase, the CP digestibility of BSF has significantly improved, increased by 12.4% in current study. Therefore, adding chitinase can effectively improve DE, ME, CP, EE, and ADF of BSF.

The ileal digestibility of AAs in BSF is an important indicator for assessing its protein nutritional value. Tan et al [32] reported that the SID values of AA for BSF vary from 76.7% to 91.8%. Crosbie et al [27] also noted that the AID values of AA for defatted BSF vary from 65.7% to 86.5%, SID values range from 79.3% to 104.6%, the AID and SID values of most AAs measured for BSF in this study were lower compared to the previous studies. Tan et al [32] used chicken manure as an organic substrate to feed BSF, which has a higher CP content than the BSF used in this study, and the ileal digestibility of AAs increased with an increase in CP content [33]. Therefore, the digestibility of AAs in Tan's study was higher than the results of this experiment. Meanwhile, several studies have indicated that the utilization of protein and AAs in BSF can be enhanced by degreasing and AA supplementation. Schiavone et al [5] found that degreasing did not have a significant impact on the ATTD of proteins. However, it improved the AID of essential and non-essential AAs. This suggests that optimizing the processing of BSF can further improve the utilization of AAs. Endogenous enzymes do not digest the β1–4 bonds between the N-acetylglucosamine subunits that compose chitin [34]. Therefore, the N within the chitin polymer and any protein encapsulated by chitin can not be digested and absorbed within the small intestine of the pig, which affected the AA digestibility of BSF [8,30]. In this study, the supplementation of chitinase significantly increased the AID of methionine, leucine, phenylalanine, isoleucine, arginine, alanine, glutamic, and proline in BSF, significantly improved the SID of methionine, tryptophan, leucine, histidine, and glutamic. As Marono et al [7] reported that the chitin content was negatively correlated with the digestibility of AA by *in vitro* trails in a single-stomached animal, the negative correlation between chitin and nutrient digestibility of BSF is likely due to the conjugation between chitin fractions and nutrients in BSF. The chitinase have hydrolyzed β-1,4-linkages of the chitin in BSF ingredients, resulting in higher AID and SID of AA of BSF. Chitin degradation by chitinase promoted to expose more CP and increasing CP digestion, which may be one of the reasons for the high digestibility of AAs in BSF after adding chitinase. This study suggested that BSF can potentially serve as a premium protein resource for pigs by adding chitinase.

The structure of an individual's diet is a crucial factor that influences the composition and function of intestinal microbiota. The present study has revealed that adding chitinase positively affects the fecal microbiota composition of growing pigs. The study found that chitinase supplementation significantly improved Shannon index, this suggests that adding chitinase increased the abundance and homogeneity of fecal microorganisms in growing pigs, this maybe because chitosan, a product obtained from chitin decomposition by chitinase, acted as a nutrient to improve intestinal microbiota [9,10]. A previous study has shown that BSF supplementation in diet enhanced the abundance of short chain fatty acid bacteria in the intestinal, which may be linked to the ability of certain bacteria to break down chitin [35]. Sarcina is a gram-positive anaerobic bacteria that associated with mucosal hyperemia and hemorrhage in animals, the decreased relative abundance of Sarcina with adding chitinase may provide benefits to intestinal health [36]. At phylum level, adding chitinase significantly increased the abundance of AcidobacteriaAcidobacteria were associated with carbohydrate metabolism, nitrogen metabolism, exopolysaccharide production and transporter functions [37], this suggests that addiing chitinase may contribute to metabolic and absorb of nutrients. The supplementation of chitinase significantly improved the abundance of *Spirochaetaceae*, *Erysipelotrichales*, *Bacilli*, *Lactobacillaceae*, and *Lactobacillales*. Research has shown that chitosan, produced after chitin degradation, can increase the relative abundance of *Lactobacillales* in mice [38]. Therefore, it is likely that the increased abundance of *Lactobacillales* with the addition of chitinase is due to degraded chitin to produce chitosan, which promoted the colonization of *Lactobacillales* [39]. *Lactobacillaceae* or *Lactobacillales* create an acidic environment in the intestine and inhibit the growth of intestinal pathogens, conferring gut health benefits on the host, and *Bacilli* can enhance intestinal barrier function and improve intestinal morphology and physical barriers in growing and fattening pigs [40,41]. Taken together, the increase in available nutrient sources produced by chitinase would afford the ability to use the diet sources more easily while also enriching bacterial diversity, and elevate the levels of beneficial bacteria. However, the precise interaction mechanism between chitinase, microbiota and digestibility of nutrients requires further investigation.

## CONCLUSION

The supplementation of 1,500 mg/kg of chitinase significantly improved the ATTD of CP, EE and ADF in the BSF, DE increased by 2.34 MJ/kg, and ME increased by 1.37 MJ/kg. Moreover, adding chitinase enhanced the AID and SID of most AAs in the BSF, such as methionine, leucine, and glutamate. Therefore, the efficiency of energy and nutrient utilization in BSF may be improved by chitinase supplementation. Chitinase have also been found to improved Shannon index and enrich bacterial diversity. It also increased the abundance of beneficial bacteria, such as *Bacilli*, *Lactobacillaceae*, and *Lactobacillales*, decreased the harmful bacteria *Sarcina* abundance. Taken together adding chitinase can regulate the composition of fecal microorganisms and improve the intestinal health of growing pigs.

## Figures and Tables

**Table 1:** Ingredient composition of experimental diets (%, as-fed basis)

Items	Experiment 1		Experiment 2	
	
	Basal diet	BSF diet	N-free diet	BSF diet
Ingredient (%)				
Corn	74.8	63.58	-	
Soybean meal	22.2	18.87	-	
Cornstarch	-	-	69.00	56.40
Sucrose	-	-	20.00	20.00
Soybean oil	-	-	3.00	
Black solider fly	-	14.58	-	20.00
Dicalcium phosphate	1.20	1.20	1.60	1.60
Cellulose acetate	-	-	4.00	-
Potassium carbonate	-	-	0.30	-
Magnesium oxide	-	-	0.10	-
Chromic oxide			0.30	0.30
Lysine	0.20	0.17	-	-
Limestone	0.80	0.80	0.90	0.90
Salt	0.30	0.30	0.30	0.30
Premix^1)^	0.50	0.50	0.50	0.50

1) Premix provided the following per kg of complete diet for growing pigs: vitamin A 5,512 IU, vitamin E 30 IU, vitamin D3 2,200 IU, vitamin K3 2.2 mg, riboflavin 4.0 mg, niacin 30.0 mg, pantothenic acid 14.0 mg, folacin 0.7 mg, choline chloride 400.0 mg, thiamine 1.5 mg, pyridoxine 3.0 mg, vitamin B12, 27.6 μg, biotin 44.0 ug, Fe 75.0 mg, Cu 100.0 mg, Zn 75.0 mg, Mn 40.0 mg, Se 0.3 mg, I 0.3 mg.

BSF, black soldier fly.

**Table 2:** Analyzed composition of black soldier fly and experimental diets used in Experiment 1 (as-fed basis)

Item	Ingredient	Diet	
	
	BSF	Basal diet	BSF diet
Dry matter (%)	96.40	90.72	91.10
Gross energy (MJ kg^−1^)	25.38	16.36	16.97
Crude protein^1)^ (%)	30.91	20.22	20.88
Ether extract (%)	40.26	1.98	6.52
NDF	35.41	18.98	15.31
ADF	19.78	6.63	5.41
Ash	10.20	4.59	5.93
Calcium	2.80	0.65	1.37
Total phosphorus	0.60	0.53	0.57

1) Crude protein = analyzed nitrogen value×(6.25×non-insects ingredients content+5.60×BSF meal content).

BSF, black soldier fly; NDF, neutral detergent fiber; ADF, acid detergent fiber.

**Table 3:** Analyzed crude protein and amino acids of black soldier fly and experimental diets used in Experiment 2

Items	Ingredient	Diet		
	
	BSF	BSF	BSF with chitinase	N-free
Crude protein^1)^ (%)	30.91	6.76	6.36	1.21
Essential amino acids (%)				-
Lysine	2.14	0.35	0.57	-
Threonine	1.30	0.30	0.30	0.01
Methionine	0.59	0.06	0.06	-
Tryptophan	0.49	0.09	0.09	-
Leucine	2.27	0.48	0.47	0.01
Valine	2.00	0.39	0.39	-
Phenylalanine	1.35	0.27	0.27	-
Isoleucine	1.45	0.29	0.29	-
Arginine	1.66	0.34	0.34	-
Histidine	1.07	0.22	0.22	-
Nonessential amino acids (%)				-
Alanine	0.00	0.45	0.45	-
Glutamic	3.89	1.00	1.00	-
Tyrosine	2.03	0.29	0.39	0.01
Serine	1.36	0.34	0.34	-
Glycine	1.71	0.40	0.40	0.02
Proline	1.89	0.42	0.43	0.01
Aspartic acid	0.00	0.70	0.70	-
Cystine	0.26	0.07	0.07	-

1) Crude protein = analyzed nitrogen value×(6.25×non-insects ingredients content+5.60×BSF meal content).

BSF, black soldier fly.

**Table 4:** Effects of chitinase on the apparent total tract digestibility of nutrients in experimental diets for growing pigs (%, as-fed basis)^1)^

Items	Basal diet	BSF diet	BSF with chitinase diet	SEM^2)^	p-value
DE (MJ/kg)	14.45	14.47	14.81	1.28	0.78
ME (MJ/kg)	14.25	14.29	14.49	0.74	0.86
DM	90.94^a^	83.79^b^	88.18^a^	1.82	0.04
CP	92.94^a^	89.99^b^	91.85^a^	1.23	0.03
EE	82.52	82.41	83.34	2.43	0.69
CF	47.24	46.56	46.97	0.91	0.83
NDF	58.46^a^	52.47^b^	52.24^b^	1.42	0.04
ADF	53.71 ^a^	48.23^b^	48.98^b^	2.82	0.02
Ash	59.72	59.55	59.93	1.20	0.74
Ca	60.25	59.91	60.38	0.73	0.94
Total P	58.73	56.87	57.12	0.70	0.52

1) BSF diets were made by replacing 15% of the energy-supplying fraction of the corn-soybean meal basal diet.

2) SEM stands for the standard error of the mean (n = 6).

a,b Values in a row with no common superscripts differ significantly (p<0.05).

BSF, black soldier fly; DE, digestible energy; ME, metabolizable energy; DM, dry matter; CP, crude protein; EE, ether extract; CF, crude fiber; NDF, neutral detergent fiber; ADF, acid detergent fiber.

**Table 5:** Effect of chitinase on energy value and the apparent total tract digestibility (ATTD, %) of nutrients of black soldier fly for growing pigs (%, as-fed basis)^1)^

Items	BSF	BSF with chitinase	SEM^2)^	p-value
Energy value				
DE (MJ/kg)	15.03^b^	17.37^a^	2.34	<0.01
ME (MJ/kg)	14.97^b^	16.34^a^	2.51	<0.01
The apparent total tract digestibility				
CP	73.27^b^	85.67^a^	2.27	<0.01
EE	81.79^b^	87.99^a^	1.18	0.04
CF	42.71	45.44	2.62	0.79
NDF	18.53	16.99	2.23	0.25
ADF	17.17^b^	22.18^a^	1.23	0.03
Ash	58.59	61.12	3.56	0.41
Ca	57.98	61.12	0.87	0.26
Total P	46.33	48.00	2.12	0.35

1) BSF diets were made by replacing 15% of the energy-supplying fraction of the corn-soybean meal basal diet.

2) SEM stands for the standard error of the mean (n = 6).

a,b Values in a row with no common superscripts differ significantly (p<0.05).

BSF, black soldier fly; DE, digestible energy; ME, metabolizable energy; CP, crude protein; EE, ether extract; CF, crude fiber; NDF, neutral detergent fiber; ADF, acid detergent fiber.

**Table 6:** Effect of the chitinase on the apparent ileal digestibility of crude protein and amino acids of BSF for growing pigs (%)^1)^

Item	BSF	BSF with chitinase	SEM^2)^	p-value
Crude protein	64.56	66.18	1.32	0.38
Essential amino acids				
Lysine	65.09	66.79	1.04	0.35
Threonine	67.90	61.88	1.34	0.26
Methionine	61.09^b^	75.46^a^	0.94	0.04
Tryptophan	74.48	75.76	1.22	0.64
Leucine	71.22^b^	75.63^a^	0.82	0.03
Valine	67.15	69.88	1.54	0.74
Phenylalanine	68.27^b^	77.01^a^	1.15	0.03
Isoleucine	63.84^b^	71.64^a^	0.36	0.04
Arginine	71.33^b^	76.36^a^	1.49	0.02
Histidine	65.50	70.83	2.54	0.50
Nonessential amino acids				
Alanine	53.33^b^	67.66^a^	1.77	0.03
Glutamic acid	46.23^b^	72.35^a^	1.41	<0.01
Tyrosine	75.27	77.84	1.50	0.44
Serine	64.32	68.00	1.28	0.23
Glycine	53.16	54.85	1.21	0.31
Proline	36.49^b^	46.45^a^	1.82	0.02
Aspartic acid	63.30	66.29	1.84	0.46
Cystine	65.71	56.61	2.43	0.64

1) SEM stands for the standard error of the mean (n = 6).

a,b Values in a row with no common superscripts differ significantly (p<0.05).

BSF, black soldier fly.

**Table 7:** Effect of the chitinase on the standardized ileal digestibility of crude protein and amino acid of BSF for growing pigs (%)^1)^

Item	BSF	BSF with chitinase	SEM^2)^	p-value
Crude protein	74.30	75.44	1.32	0.38
Essential amino acids				
Lysine	74.65	76.63	1.04	0.35
Threonine	70.75	71.88	1.34	0.76
Methionine	80.18^b^	85.85^a^	0.94	0.04
Tryptophan	73.49^b^	84.00^a^	1.22	0.03
Leucine	80.68^b^	85.71^a^	0.82	0.02
Valine	76.54	79.91	1.54	0.74
Phenylalanine	87.64	87.04	1.15	0.73
Isoleucine	73.24	81.73	0.36	0.62
Arginine	80.24	86.05	1.49	0.24
Histidine	70.12^b^	80.64^a^	2.54	0.03
Nonessential amino acids				
Alanine	72.50	77.94	1.77	0.45
Glutamic acid	55.65^b^	82.56^a^	1.41	0.03
Tyrosine	85.23	87.41	1.50	0.55
Serine	72.15	78.06	1.28	0.62
Glycine	74.87	76.08	1.21	0.39
Proline	45.49	54.87	1.82	0.41
Aspartic acid	74.65	76.63	1.84	0.35
Cystine	72.75	71.88	2.43	0.76

1) Values for SID were calculated by correcting the apparent ileal digestibility values with the basal endogenous losses. Basal endogenous losses (gkg^−1^ dry matter intake) averaged as arginine, 0.54; histidine, 0.18; isoleucine, 0.30; leucine, 0.35; lysine, 0.26; methionine, 0.09; phenylalanine, 0.27; threonine, 0.32; tryptophan, 0.12; valine, 0.34; alanine, 0.49; asparticacid, 0.48; cystine, 0.13; glutamicacid, 0.54; glycine, 1.36; serine, 0.38; tyrosine, 0.15.

2) SEM stands for the standard error of the mean (n = 6).

a,b Values in a row with no common superscripts differ significantly (p<0.05).

BSF, black soldier fly; SID, standardized ileal digestibility.
